# Increased Frequencies of Switched Memory B Cells and Plasmablasts in Peripheral Blood from Patients with ANCA-Associated Vasculitis

**DOI:** 10.1155/2020/8209737

**Published:** 2020-11-28

**Authors:** Evelina Elmér, Sofia Smargianaki, Åsa Pettersson, Lillemor Skattum, Sophie Ohlsson, Thomas Hellmark, Åsa C. M. Johansson

**Affiliations:** ^1^Lund University, Skåne University Hospital, Department of Laboratory Medicine, Haematology and Transfusion Medicine, and Clinical Immunology and Transfusion Medicine, Region Skåne, Lund, Sweden; ^2^Lund University, Skåne University Hospital, Department of Clinical Sciences Lund, Nephrology, Lund, Sweden; ^3^Clinical Immunology and Transfusion Medicine, Region Skåne, and Department of Laboratory Medicine, Microbiology, Immunology and Glycobiology, Lund University, Lund, Sweden; ^4^Lund University, Skåne University Hospital, Department of Laboratory Medicine, Haematology and Transfusion Medicine, and Clinical Genetics and Pathology, Region Skåne, Lund, Sweden

## Abstract

B cells are thought to play a central role in the pathogenesis of antineutrophil cytoplasmic antibody- (ANCA-) associated vasculitis (AAV). ANCAs have been proposed to cause vasculitis by activating primed neutrophils to damage small blood vessels. We studied a cohort of AAV patients of which a majority were in remission and diagnosed with granulomatosis with polyangiitis (GPA). Using flow cytometry, the frequencies of CD19^+^ B cells and subsets in peripheral blood from 106 patients with AAV and 134 healthy controls were assessed. B cells were divided into naive, preswitch memory, switched memory, and exhausted memory cells. Naive and switched memory cells were further subdivided into transitional cells and plasmablasts, respectively. In addition, serum concentrations of immunoglobulin A, G, and M were measured and clinical data were retrieved. AAV patients displayed, in relation to healthy controls, a decreased frequency of B cells of lymphocytes (5.1% vs. 8.3%) and total B cell number. For the subsets, a decrease in percentage of transitional B cells (0.7% vs. 4.4%) and expansions of switched memory B cells (22.3% vs. 16.5%) and plasmablasts (0.9% vs. 0.3%) were seen. A higher proportion of B cells was activated (CD95^+^) in patients (20.6% vs. 10.3%), and immunoglobulin levels were largely unaltered. No differences in B cell frequencies between patients in active disease and remission were observed. Patients in remission with a tendency to relapse had, compared to nonrelapsing patients, decreased frequencies of B cells (3.5% vs. 6.5%) and transitional B cells (0.1% vs. 1.1%) and an increased frequency of activated exhausted memory B cells (30.8% vs. 22.3%). AAV patients exhibit specific changes in frequencies of CD19^+^ B cells and their subsets in peripheral blood. These alterations could contribute to the autoantibody-driven inflammatory process in AAV.

## 1. Introduction

Antineutrophil cytoplasmic antibody- (ANCA-) associated vasculitis (AAV) is a group of uncommon autoimmune disorders characterized by inflammation and destruction of predominantly small blood vessels and the presence of circulating ANCA [1]. Clinical disease phenotypes include eosinophilic granulomatosis with polyangiitis (EGPA), granulomatosis with polyangiitis (GPA), and microscopic polyangiitis (MPA) [2]. ANCAs are autoantibodies directed against cytoplasmic antigens, primarily proteinase 3 (PR3) and myeloperoxidase (MPO), found in the primary granules of neutrophils and in the lysosomes of monocytes. PR3-ANCA is associated with GPA (75%), whereas MPO-ANCA is more commonly associated with MPA (60%). ANCAs are present in approximately 50% of patients with EGPA, typically MPO-ANCA [1, 3].

The majority of AAV patients have renal involvement in terms of rapidly progressing glomerulonephritis. There is no curative treatment, but current therapy has transformed AAV from a fatal disease to a chronic illness with relapsing course and limited morbidity. The pathogenesis is multifactorial and influenced by genetics, environmental factors, and responses of the innate and adaptive immune system [4]. ANCAs have been proposed to cause vasculitis by activating primed neutrophils to damage small blood vessels [5].

As precursors of antibody-secreting plasma cells, B cells have a central role in the pathogenesis of AAV [6]. In addition, B cells can act as antigen-presenting cells and hence initiate T cell responses by providing costimulatory signals and secrete cytokines and growth factors [7]. B cells regulate immunological functions by suppressing T cell proliferation and producing proinflammatory cytokines, such as interferon-*γ*, tumor necrosis factor-*α*, and interleukin-17 [8]. Further, the efficacy of B cell depletion therapy, e.g., rituximab, in AAV supports the importance of B cells in the pathogenesis. Rituximab has been shown to be as effective as cyclophosphamide treatment in inducing remission in severe AAV and possibly superior in relapsing disease [9, 10]. The return of B cells and ANCA positivity after rituximab treatment may predict relapse of AAV [11].

The B cell-activating factor (BAFF), also known as B Lymphocyte Stimulator (BLyS), is a positive regulator of B cell survival, differentiation, and proliferation and has been associated with autoimmunity. Sanders et al. found that plasma levels of BAFF were elevated in patients with AAV. The levels were not affected by disease activity or ANCA status [12]. Matsumoto et al. have shown that AAV patients display higher proportions of plasma cells and plasmablasts as compared to healthy controls. In addition, immune cell phenotyping was similar between patients with MPA, GPA, and EGPA [13]. Recently, von Borstel et al. demonstrated that an increased frequency of circulating plasmablasts and plasma cells (CD27^+^CD38^++^ B cells) in GPA patients during remission is related to a higher relapse risk [14]. Furthermore, the percentage of activated B cells in GPA has been shown to correlate with disease activity [15].

We hypothesized that AAV patients have altered frequencies of B cell subsets and that the alterations correlate with disease activity and/or tendency to relapse. Based on previously published observations, our primary objectives were to study transitional, naive, and memory B cell subsets and B cell count in peripheral blood. In addition, we explored if activated B cells/subsets and immunoglobulin levels correlated with disease activity and/or tendency to relapse.

## 2. Materials and Methods

### 2.1. Patients and Controls

149 patients with AAV attending or referred to the outpatient clinics of Nephrology and Rheumatology, Skåne University Hospital, Lund, Sweden, were consecutively included from October 2011 to January 2019. The diagnosis was determined using the algorithm described by Watts et al. [16]. Patient blood samples were collected at diagnosis when possible and at follow-up visits.

Patients with known malignancy, ongoing infection, or coexisting autoimmune disorder were not included. Further, patients treated with rituximab (*n* = 27), in dialysis (*n* = 6), or less than 500 CD19^+^ cells within the lymphocyte population (*n* = 8) were excluded. Two patients were excluded due to lack of B cell data because of technical problems. For the remaining 106, one sample was analyzed per patient, usually the last that did not meet any of the exclusion criteria. Patient characteristics and demographics are described in Table 1.

Blood samples were also acquired from 134 healthy blood donors (HBD) at the blood donor central in Lund. There was no age or gender matching between the patients and HBD. The study was approved by the regional ethical review board in Lund, Sweden (permit number 2008/110). Prior to inclusion, all subjects gave written informed consent.

### 2.2. Measurements and Clinical Parameters

Data on disease activity, date of diagnosis, white blood cell count (WBC), C-reactive protein (CRP), creatinine in plasma, ANCA serology, tendency to relapse, date of latest relapse, dialysis, and medication were obtained from medical records. Disease activity was estimated according to the Birmingham Vasculitis Activity Score version 3 (BVAS3) [17]. Active disease was defined as BVAS3 ≥ 2 and remission as BVAS3 ≤ 1. Tendency to relapse was defined as recurrence of disease after complete remission had been achieved, in patients who had at least one year of follow-up and had received standard of care. Recurrence of the disease was defined as BVAS3 ≥ 1 in combination with increased immunosuppressive therapy. WBC, CRP, and creatinine in plasma were analyzed as routine clinical samples at Clinical Chemistry, Region Skåne, Lund. Serum levels of PR3-ANCA and MPO-ANCA were measured with ELISA including a capture technique at Wieslab (Svar Life Science AB, Malmö, Sweden) and at Clinical Immunology and Transfusion Medicine, Region Skåne, Lund. The estimated glomerular filtration rate (eGFR) was calculated for AAV patients using the CKD-EPI creatinine (2009) equation (http://www.mdrd.com). Immunoglobulins were analyzed as routine clinical samples at Clinical Immunology and Transfusion Medicine, Region Skåne, Lund.

### 2.3. Phenotypic Characterization of B Cells

Peripheral blood samples were collected from patients and HBD in heparin tubes (BD Vacutainer ref 369622) and stored at room temperature and protected from light until analyzed (within 24 h). The expression of selected surface markers on B cells was analyzed using flow cytometry. Briefly, peripheral blood was lysed using 0.84% NH_4_Cl. An antibody cocktail of the following monoclonal fluorescent-labeled antibodies (BD Biosciences, San Jose, CA, USA) was added to the suspension of leukocytes: CD19 PerCP Cy5.5 (HIB19), IgD V450 (IA6-2), CD27 APC H7 (M-T271), CD38 PE Cy7 (HB-7), CD24 FITC (ML5), CD95 APC (DX2), and CD45 V500 (HI30).

Acquisition was performed on a FACSCanto II flow cytometer with the accompanying FACSDiva software (Becton Dickinson, Franklin Lakes, NJ, USA). Data were analyzed using Kaluza Analysis Software version 2.1 (Beckman Coulter, Brea, CA, USA). Doublet cells were excluded by plotting forward scatter height against forward scatter area, and single cells were divided into monocytes, lymphocytes, and granulocytes based on forward and side scatter properties. At least 50.000 lymphocytes were acquired. The phenotypes of immune cell subsets were defined based on the Human Immunology Project protocol [18]. Details of the gating strategy are shown in Figure S1. Analysis using this panel allowed identification of B cells (CD19^+^ lymphocytes) and its subsets: naive B cells (CD19^+^CD27^−^IgD^+^), preswitch memory B cells (CD19^+^CD27^+^IgD^+^), switched memory B cells (CD19^+^CD27^+^IgD^−^), exhausted memory B cells (CD19^+^CD27^−^IgD^−^), transitional B cells (CD19^+^CD27^−^IgD^+^CD24^+^CD38^++^), and plasmablasts (CD19^+^CD27^+^IgD^−^CD24^−^CD38^++^). The percentages of activated (CD95^+^) B cells, naive B cells, preswitch memory B cells, switched memory B cells, and exhausted memory B cells of the total number of CD19^+^ lymphocytes were analyzed. Patients and controls were analyzed with flow cytometry in parallel. All values are given as a percentage of CD19^+^ B cells if not otherwise specified. Absolute numbers of B cell subsets were based on the proportion (%) of B cells within the lymphocyte population combined with the absolute number of lymphocytes from the WBC count.

No absolute numbers of lymphocytes were available for HBD. However, a reference material of 50 healthy blood donors was collected at Clinical Immunology and Transfusion Medicine, Region Skåne, Lund in 2013 for B cells, giving a range of CD19^+^ B cells (of lymphocytes) of 5.5-20% (70-460 10^6^/L).

### 2.4. Statistical Analysis

Statistical analyses were performed with GraphPad Prism 8.4.2 software (GraphPad Software, San Diego, CA, USA). The Mann-Whitney *U* test was used for two-group comparisons and Kruskal-Wallis with Dunn's multiple comparisons test for three or more groups. Correlations were determined by Spearman's correlation test and linear regression analysis. Statistical analysis was only performed for the parameters considered relevant for answering one or more of the hypotheses of the study. Samples with CRP concentrations below the lower limit of detection (<0.6 mg/L, *n* = 13) and IgA (<0.07 g/L, *n* = 2) were set to 0. Subgroups of *n* < 5 patients were not included in the statistical analyses. Values are expressed as median with interquartile range (IQR) unless otherwise specified. Results were considered statistically significant at *p* < 0.05.

## 3. Results

### 3.1. Patient Characteristics

The clinical and demographic characteristics of the patients with AAV (*n* = 106) at the time of sampling are reported in Table 1. The median age was 70.0 years (57.5-75.3), and the female to male ratio was 1.12 to 1.0. The majority of patients were diagnosed with GPA (60%), whereas patients with MPA and EGPA represented 33% and 7% of the cohort, respectively. Most of the patients were in remission (77%), and 23% had disease activity with BVAS3 ≥ 2 (median score 6, range 2-26). A tendency to relapse was observed in 36% of the patients, and for those in remission at the time of sampling, the median time since the onset of the latest relapse was 43.3 months (12.6-144). Thirty-four percent of the patients had no tendency to relapse, and in 30%, it could not be decided according to the definition, e.g., too short follow-up period or onset of disease at the time of sampling.

At the time of sampling, 53% of the patients were treated with prednisolone (dose 10.0 mg/day, 5.00-15.0), 31% were treated with azathioprine (100 mg/day, 75.0-138), 8.5% were treated with methotrexate (25.0 mg/week, 17.5-25.0), 5.7% were treated with mycophenolate mofetil (2000 mg/day, 1313-2125), 10.4% were treated with cyclophosphamide infusion, and 24.5% had no medication.

Demographic data on age and gender were available for 102 of the 134 healthy controls, giving a median age of 49 years (range 19–74) and a female to male ratio of 1.1 to 1.0.

### 3.2. Decreased Frequencies of B Cells and Transitional B Cells in AAV Patients

**Figure 1:**
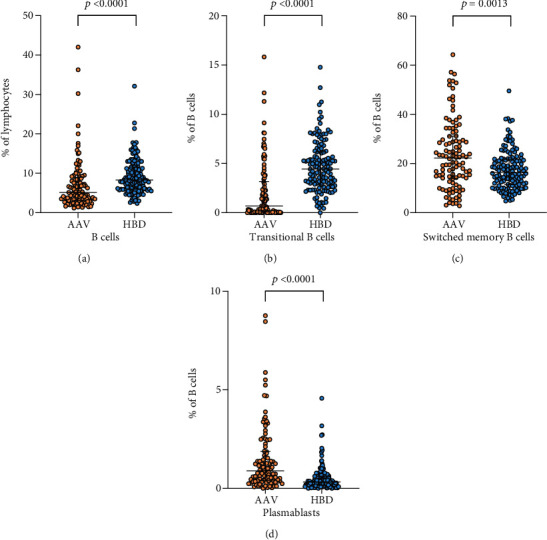
Comparisons of B cells and subsets between vasculitis patients and healthy blood donors. (a) Percentage of CD19^+^ B cells of lymphocytes, (b) percentage of transitional B cells (of CD19^+^ B cells), (c) percentage of switched memory B cells (of CD19^+^ B cells), and (d) percentage of plasmablasts (of CD19^+^ B cells), in peripheral blood from patients with antineutrophil cytoplasmic autoantibody- (ANCA-) associated vasculitis (AAV, *n* = 106) and healthy blood donors (HBD, *n* = 134). The Mann-Whitney test was used to calculate the level of significance. Data are presented with medians and interquartile ranges. In figure (d), one data point (20.56) in the AAV group is not shown for presentation purposes but included in statistical calculations.

To investigate differences in the distribution of B cells (CD19^+^ lymphocytes) and its subsets between AAV patients and HBD, the percentages of activated (CD95^+^) B cells, naive B cells (CD19^+^CD27^−^IgD^+^), preswitch memory B cells (CD19^+^CD27^+^IgD^+^), switched memory B cells (CD19^+^CD27^+^IgD^−^), exhausted memory B cells (CD19^+^CD27^−^IgD^−^), transitional B cells (CD19^+^CD27^−^IgD^+^CD24^+^CD38^++^), and plasmablasts (CD19^+^CD27^+^IgD^+^CD24^−^CD38^++^) of the total number of CD19^+^ lymphocytes were analyzed. The AAV patients had a lower percentage of CD19^+^ B cells of lymphocytes compared to HBD (*p* < 0.0001, Figure 1(a), Table 2) and a lower percentage of transitional B cells (*p* < 0.0001, Figure 1(b), Table 2). The absolute number of B cells was lower in AAV patients (26.5 10^6^/L, 15.2-69.4) (Table 2) compared to a separate reference material of 50 healthy blood donors (70-460 10^6^/L).

### 3.3. Increased Frequencies of Switched Memory B Cells and Plasmablasts in AAV Patients

Memory B cells can be defined by CD27, although some subsets, generally associated with exhausted B cells, do not express CD27. Switched memory B cells have undergone class-switching (do not express IgD) and are indicators of normal B cell activation and development in germinal centers in lymph nodes or other secondary lymphoid tissues.

The percentage of switched memory B cells was higher in patients compared to HBD (*p* = 0.0013, Figure 1(c), Table 2).

Plasmablast refers to a short-lived differentiation stage between a postgerminal center B cell and a mature plasma cell, the latter specialized to produce single isotype antibodies. The patients had a higher percentage of plasmablasts compared to HBD (*p* < 0.0001, Figure 1(d), Table 2).

### 3.4. Largely Unaltered Immunoglobulin Levels in AAV Patients

Since the percentage of plasmablasts, the precursors of antibody-producing plasma cells, was higher in AAV patients, we measured the levels of immunoglobulins in serum. The levels of immunoglobulins (IgA, IgG, and IgM) in AAV patients were within the reference range (Table 3), except for IgA in the EGPA group where the lower IQR was below the reference range. Two patients diagnosed with MPA had IgA deficiency. AAV patients with a tendency to relapse had lower IgG levels compared to AAV patients with no tendency to relapse (*p* = 0.0446). Patients with medication had lower IgG levels compared to patients without medication (*p* = 0.0021).

Overall, there were no correlations between immunoglobulin levels and plasmablasts in AAV patients, neither for percentage nor for absolute concentration of plasmablasts, except for a weak but significant correlation between IgG and the concentration of plasmablasts (*r*^2^ = 0.044, *p* = 0.0494).

When analyzing only patients in remission, there was a correlation between IgG and the concentration of plasmablasts (*r*^2^ = 0.132, *p* = 0.0023). Moreover, there were no correlations between immunoglobulin levels and the frequency or concentration of plasmablasts in patients in active disease, patients with or without a tendency to relapse, or patients with and without medication (data not shown).

### 3.5. Increased Frequencies of CD95^+^ B Cells and Subsets in AAV Patients

**Figure 2:**
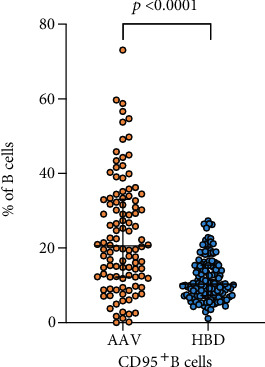
Comparison of CD95^+^ B cells (of CD19^+^ B cells) in peripheral blood from patients with antineutrophil cytoplasmic autoantibody- (ANCA-) associated vasculitis (AAV, *n* = 106) and healthy blood donors (HBD, *n* = 134). The Mann-Whitney test was used to calculate the level of significance. Data are presented with medians and interquartile ranges.

The Fas receptor, CD95, has been suggested to be a marker of B cell activation. The percentage of CD95^+^ B cells was increased in patients compared to HBD (*p* < 0.0001, Figure 2, Table 2). In all analyzed B cell subsets, the percentage of CD95^+^ cells (of the respective subset) was higher compared to HBD (Table 2).

### 3.6. No Differences in B Cell Frequencies in Active and Inactive Disease

To characterize B cell subset distribution in relation to disease activity, patients were divided into two groups: active disease (BVAS3 ≥ 2) or remission (BVAS3 ≤ 1). Most of the AAV patients were in remission (*n* = 82). There were no differences in the frequencies of CD19^+^ B cells of lymphocytes, in its subsets, or in the absolute number of B cells between patients in active disease and patients in remission (Table 4).

### 3.7. Decreased Frequencies of B Cells and Transitional B Cells in Patients with a Tendency to Relapse

To characterize B cell subset distribution in relation to tendency to relapse (defined in Materials and Methods), patients were divided into two groups. Patients with a tendency to relapse (*n* = 38) had a lower frequency of B cells of lymphocytes (3.82%, 2.63-6.66) compared to patients with no tendency to relapse (*n* = 36) (6.53%, 3.68-9.21, *p* = 0.0215). Nine of the patients in the relapse group had an active disease, and the remaining were in remission (*n* = 29). In the group without a tendency to relapse, all patients were in remission.

**Figure 3:**
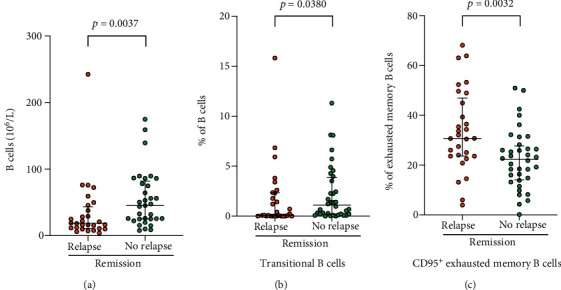
Comparisons of B cells and subsets between patients in remission with and without a tendency to relapse. (a) Concentration of CD19^+^ B cells in peripheral blood from patients with antineutrophil cytoplasmic autoantibody- (ANCA-) associated vasculitis (AAV) in remission with (*n* = 28) and without (*n* = 33) tendency to relapse. (b) Percentage of transitional B cells (of CD19^+^ B cells), (c) percentage of CD95^+^ exhausted memory B cells (of exhausted memory B cells), in peripheral blood from patients with AAV in remission with (*n* = 29) and without (*n* = 36) tendency to relapse. The Mann-Whitney test was used to calculate the level of significance. Data are presented with medians and interquartile ranges.

When comparing only patients in remission, patients with a tendency to relapse had a lower frequency of B cells compared to patients with no tendency to relapse (*p* = 0.0295, Table 5). This was also reflected in the absolute number of B cells (Figure 3(a), Table 5). The frequency of transitional B cells was lower in patients with a tendency to relapse (*p* = 0.0380, Figure 3(b), Table 5). There was also a higher frequency of CD95^+^ exhausted memory B cells (of exhausted memory B cells) in the patients in remission with a tendency to relapse (*p* = 0.0032, Figure 3(c), Table 5).

The immunomodulating medication for patients in remission is listed in Table 5. 42% of patients in remission with no tendency to relapse had no medication at the time of sampling; the corresponding figure for patients with tendency was 7%. There were no significant differences in doses of medications between the groups.

### 3.8. Decreased Frequency of B Cells in Patients with Immunomodulating Medication

To broadly report the immunomodulating medication, patients were divided into two groups. AAV patients with ≥1 of the following medications (75.5%, of which 74% were in remission): prednisolone, azathioprine, methotrexate, mycophenolate mofetil, cyclophosphamide, were compared to patients with none of the five medications (24.5%, 88% were in remission). BVAS3 did not differ between the groups (Table S1). Patients with pharmacological treatment had a lower percentage of B cells compared to patients without medications (*p* < 0.0001), which was reflected in the absolute B cell count (*p* < 0.0001) and in B cell subsets (Table S1).

## 4. Discussion

B cells are thought to have a central role in the pathogenesis of antineutrophil cytoplasmic antibody- (ANCA-) associated vasculitis (AAV), a disease characterized by autoantibodies and effectively treated by B cell depletion using monoclonal antibodies directed against the B cell antigen CD20.

In this study, patients with AAV had a lower percentage and absolute number of CD19^+^ B cells. This is in line with previous studies on GPA patients in remission, demonstrating that patients have lower absolute numbers of circulating CD19^+^ B cells [19] and lower B cell frequency [14]. Lepse et al. on the other hand, found no difference in the percentage of CD19^+^ B cells between AAV patients and healthy controls [20]. The discrepancy between the studies could possibly be related to differences in medication or disease activity between the cohorts.

Transitional B cells are immature B cells and are related to interleukin 10- (IL-10-) producing regulatory B cells (Bregs) in terms of phenotypical and functional similarities [6]. However, the overlap between transitional B cells and Bregs is debated. Transitional B cells can also produce IL-10 and regulate CD4^+^ T cell proliferation and differentiation toward T helper effector cells [21].

We found that the patients had a lower proportion of transitional B cells. This is in contrast to von Borstel et al. that found no difference in transitional B cell frequencies between GPA patients with future relapse, nonrelapsing patients, and healthy controls [14]. Partly in agreement with our finding, Lepse et al. showed that AAV patients in active disease had a decreased percentage of transitional B cells compared to patients in remission and healthy controls [20]. In further support of our findings, a low frequency of transitional B cells has been noted in neuroimmunological diseases, including multiple sclerosis (MS) [22] and neuromyelitis optica (NMO) [23]. However, the frequency of CD24^hi^CD38^hi^ transitional B cells is elevated in patients with systemic lupus erythematosus (SLE) and Sjögren's syndrome (SS) [24].

Memory B cells are optimized to interact with T cells and to yield strong antibody responses. Autoreactive antibodies likely contribute to the chronic and progressive course typically observed in autoimmune disorders [25]. High frequencies of memory B cells are associated with poor clinical response to rituximab (anti-CD20) treatment [26]. We found an increased frequency of switched memory B cells in AAV patients, and patients with medication had a higher percentage of switched memory B cells compared to the nonmedication group and healthy controls.

Previously published observations have reported a decreased proportion of circulating CD27^+^ memory B cells in AAV patients [14, 19, 20, 27]. Two of the studies also showed that the AAV patients had an increased percentage of naive B cells [14, 20], whereas we found a decreased percentage of naive B cells in the patient cohort. The discrepancy could likely be attributed to differences in medication or disease severity as we found that AAV patients without medication do not display a decreased proportion of naive B cells. In support of our finding, patients with hepatitis C-related mixed cryoglobulinemia vasculitis had a decreased percentage of naive B cells and increased frequencies of memory B cells and plasmablasts compared to healthy and hepatitis C virus controls [28].

In the present study, none of the patients had received anti-B cell treatment and they all had immunoglobulin levels (total IgA, IgG, and IgM) within the reference range, except for IgA in the EGPA group where the lower IQR was below the reference range. We observed lower concentrations of IgG in patients with immunosuppressive medication and in those with a tendency to relapse. The difference in IgG levels between the relapse and nonrelapse groups could possibly be a result of differences in medication, since a higher proportion of patients in the relapse group had medication. It has been reported that 10-13% of patients with multisystem autoimmune disease have low IgG, IgM, and IgA and that the IgG levels are lower in patients who have received cyclophosphamide [29]. Another study on patients with autoimmune disease (the majority diagnosed with GPA) has shown that the total dose of cyclophosphamide is not associated with the IgG concentration [30].

Multiple biomarkers of disease activity in AAV have been proposed, including platelet count [31], lung involvement [32], and prognostic nutritional index [33]. A rise in or persistence of ANCAs during remission is only modestly predictive of future disease relapse [34]. A low percentage of circulating CD5^+^ B cells, which partially overlaps with the immunophenotype of Bregs, has been shown to correlate with disease activity and a shorter time to relapse after rituximab treatment in AAV [35]. Further, temporal alterations in CD14^++^CD16^+^ intermediate monocyte counts have been associated with disease relapse in AAV patients [36].

The percentage of circulating plasmablasts and plasma cells (CD27^+^CD38^++^ B cells) has been shown to be increased in GPA patients with future relapse [14]. Increased frequency of circulating CD27^+^CD38^++^ B cells during remission could therefore be a potential marker to identify patients at risk of relapse. Also, in other autoimmune diseases such as SLE [37, 38] and IgG4-related disease [39], the plasmablast frequency has been reported to be related to disease activity. Here, we report that the AAV patients had a higher percentage of plasmablasts compared to healthy controls, but there was no difference between patients in active versus inactive disease, or between the relapse versus no-relapse group.

The Fas death receptor, CD95, is upregulated on B cells upon activation. We observed an increased frequency of activated (CD95^+^) B cells in AAV patients, in B cells in total and in all analyzed B cell subsets, compared to healthy controls. In a previous study of GPA, B cell activation was related to active disease, whereas T cell activation persisted during remission [15]. In addition, the percentage of CD38^bright^ B cells was higher in patients with generalized active disease compared to patients in less active disease groups and healthy controls. We observed no differences in the proportion of CD95^+^ B cells between patients in active and inactive disease. The percentage of CD95^+^ B cells was higher in patients with medication compared to patients without medication and to healthy controls. This was reflected in all subsets of B cells except for CD95^+^ switched memory B cells where both patients with and without medication displayed an increased frequency of similar magnitude.

While alterations of B cell subsets have been described in multiple autoimmune diseases, it remains unknown whether a specific B cell subset profile is associated with disease relapse. In our observations of patients in remission, the patients with a tendency to relapse had lower B cell frequency and lower B cell count compared to patients without relapsing disease. This may be a result of immunosuppressive treatment as a higher proportion of patients in the relapse group had medication. In line with this, we show that patients with immunosuppressants had lower percentage and lower B cell count compared to the nonmedication group, whereas there was no difference between patients without immunosuppressants and healthy controls. In agreement with our findings, Appelgren et al. found that the prednisolone dose correlated negatively with the absolute number of B cells and the number of naive and memory B cells (but not exhausted memory B cells) [27]. Treatment with cyclophosphamide has been shown to reduce B cell counts, albeit the rate and magnitude of the decrease are less than with rituximab [10].

Patients in the relapse group displayed no difference in the frequency of exhausted memory B cells as compared to the nonrelapse group; however, the relapse group had a higher percentage of CD95^+^ exhausted memory B cells. In SLE, a specific population of exhausted memory B cells has been demonstrated to be highly enriched, which implicates these autoreactive cells in autoimmune disease [40].

Limitations of this study include heterogeneity of disease duration, type of vasculitis diagnosis, lack of timing of blood collection (samples were obtained from patients at routine clinical visits), that no absolute numbers of lymphocytes were available for the healthy blood donors, and that they were not age- or gender-matched. An additional important limitation is the possible influence of medications on disease severity and the hematopoietic system. To investigate whether the alterations in B cell subsets are specifically related to the immunosuppressive treatment or the autoimmune disease (or both), studies including age- and gender-matched therapy controls are required.

## 5. Conclusions

In the present study, we primarily investigated if patients with antineutrophil cytoplasmic antibody- (ANCA-) associated vasculitis have altered frequencies of B cells and subsets in peripheral blood. As the main findings, the patient cohort displayed both a decreased frequency of B cells (of lymphocytes) and total B cell number, as well as specific changes in frequencies of B cell subsets. There was an expansion of switched memory B cells as well as plasmablasts and a decrease in the percentage of transitional B cells. Patients with a tendency to relapse had decreased frequencies of B cells and transitional B cells, as well as a higher proportion of activated exhausted memory B cells, compared to patients without relapsing disease. B cells can exert both regulatory and effector functions. Alterations in B cell subsets could translate to changes in the balance of these functions and may contribute to the autoantibody-driven inflammatory process, influence disease activity, and risk of relapse. However, the relative influence of disease activity and effect of medication on the B cell phenotype is difficult to separate in a complex autoimmune disease such as vasculitis.

## Figures and Tables

**Table 1:** Patient characteristics and demographics.

	GPA (*n* = 64)	MPA (*n* = 35)	EGPA (*n* = 7)
Age, years, median (IQR)	67.0 (55.0-73.8)	73.0 (63.0-82.0)	71.0 (56.0-76.0)
Female/male, *n* (%)	26 (41)/38 (59)	19 (54)/16 (46)	5 (71)/2 (29)
Age at diagnosis, years, median (IQR)	50.5 (37.3-66.0)	68.0 (60.0-75.0)	66.0 (38.0-71.0)
Disease duration, years, median (IQR)	6.74 (3.59-17.8)	2.21 (0.447-9.95)	7.91 (4.82-18.0)
ANCA specificity, *n* (%)			
PR3	45 (70)	2 (6)	0 (0)
MPO	17 (27)	30 (86)	3 (43)
PR3 and MPO	0 (0)	1 (3)	0 (0)
No ANCA	1 (1.5)	1 (3)	3 (43)
Data not available	1 (1.5)	1 (3)	1 (14)
Disease activity			
Active disease, *n* (%)	14 (22)	9 (26)	1 (14)
BVAS3, median (range)	6 (2-26)	14 (5-21)	4
Remission, *n* (%)	50 (78)	26 (74)	6 (86)
Tendency to relapse, *n* (%)			
Yes	29 (45)	8 (23)	1 (14)
Time since onset of the latest relapse, months, median (IQR)^a^	66.5 (24.5-178)	8.30 (4.73-26.8)	NA
No	18 (28)	13 (37)	5 (71)
Not applicable	17 (27)	14 (40)	1 (14)
WBC, 10^9^/L, median (IQR)^b^	6.55 (5.10-8.45)	7.70 (5.65-9.40)	7.40 (5.48-10.1)
P-CRP, mg/L, median (IQR)^c^	2.25 (1.10-4.55)	6.70 (2.00-14.5)	0.00 (0.00-3.23)
P-creatinine, *μ*mol/L, median (IQR)^d^	102 (80.0-136)	137 (101-193)	84.5 (62.8-108)
eGFR, mL/min/1.73 m^2^, median (IOR)	58.0 (40.0-80.0)	39.0 (21.0-51.0)	69.0 (52.8-88.0)
Medication, *n* (%), dose, median (IQR)			
Prednisolone, mg/day	30 (47) 6.88 (5.00-13.1)	22 (63) 10.0 (8.75-31.3)	4 (57) 5.00 (2.13-16.9)
Azathioprine, mg/day	16 (25) 100 (75.0-144)	13 (37) 100 (75.0-100)	4 (57) 125 (100-188)
Methotrexate, mg/week	9 (14) 25.0 (17.5-25.0)	0 (0)	0 (0)
Mycophenolate mofetil, mg/day	6 (9) 2000 (1313-2125)	0 (0)	0 (0)
Cyclophosphamide	4 (6)	7 (20)	0 (0)
No medication	18 (28)	6 (17)	2 (29)

GPA: granulomatosis with polyangiitis; MPA: microscopic polyangiitis; EGPA: eosinophilic granulomatosis with polyangiitis; IQR: interquartile range; ANCA: antineutrophil cytoplasmic autoantibodies; PR3: proteinase 3; MPO: myeloperoxidase; BVAS3: Birmingham Vasculitis Activity Score version 3; NA: not applicable; WBC: white blood cell; CRP: C-reactive protein; eGFR: estimated glomerular filtration rate. ^a^For those in remission at the time of sampling. *n* = 19 (GPA), *n* = 6 (MPA). ^b^Reference range 3.5-8.8 10^9^/L. ^c^Reference range < 0.6 mg/L. ^d^Reference range 60-105 *μ*mol/L (male), 45-90 *μ*mol/L (female). For BVAS3, WBC, P-CRP, P-creatinine, and eGFR, the cohort sizes were *n* = 62‐64 (GPA), *n* = 33‐35 (MPA), and *n* = 6‐7 (EGPA).

**Table 2:** Frequencies of B cells and subsets in patients with AAV and HBD.

Phenotype	Patients (AAV) (*n* = 106)	HBD (*n* = 134)	*p* value
B cells (% of lymphocytes)	5.13 (3.15-8.98)	8.33 (5.98-11.6)	<0.0001
B cells (10^6^/L)^a^	26.5 (15.2-69.4)		
B cell subsets			
Naive (% of B cells)	47.8 (33.4-60.3)	56.7 (47.9-67.0)	0.0002
Naive (10^6^/L)	12.1 (4.49-33.9)		
Preswitch memory (% of B cells)	6.32 (3.63-10.1)	7.00 (4.65-10.2)	ns
Preswitch memory (10^6^/L)	1.86 (0.818-4.28)		
Switched memory (% of B cells)	22.3 (14.0-31.1)	16.5 (11.4-21.8)	0.0013
Switched memory (10^6^/L)	5.78 (2.98-11.2)		
Exhausted memory (% of B cells)	21.2 (14.1-25.9)	17.4 (12.0-21.6)	0.0006
Exhausted memory (10^6^/L)	5.30 (2.69-15.1)		
Transitional (% of B cells)	0.695 (0.0700-3.17)	4.44 (2.83-6.18)	<0.0001
Transitional (10^6^/L)	0.278 (0.0107-1.29)		
Plasmablasts (% of B cells)	0.885 (0.390-1.87)	0.320 (0.160-0.613)	<0.0001
Plasmablasts (10^6^/L)	0.293 (0.138-0.587)		
Activated B cells and subsets			
CD95^+^ B cells (% of B cells)	20.6 (12.3-33.1)	10.3 (7.24-15.5)	<0.0001
CD95^+^ B cells (10^6^/L)	5.48 (3.24-11.2)		
CD95^+^ naive (% of naive)	3.38 (1.49-7.24)	1.02 (0.575-1.71)	<0.0001
CD95^+^ naive (10^6^/L)	0.427 (0.194-0.833)		
CD95^+^ preswitch memory (% of preswitch memory)	23.0 (16.4-38.7)	13.5 (9.07-19.8)	<0.0001
CD95^+^ preswitch memory (10^6^/L)	0.355 (0.190-0.892)		
CD95^+^ switched memory (% of switched memory)	59.7 (51.2-73.0)	44.6 (36.0-51.9)	<0.0001
CD95^+^ switched memory (10^6^/L)	3.15 (1.70-6.66)		
CD95^+^ exhausted memory (% of exhausted memory)	25.2 (15.0-35.0)	14.0 (8.38-18.2)	<0.0001
CD95^+^ exhausted memory (10^6^/L)	1.27 (0.667-2.78)		

Frequencies of B cells (CD19^+^ lymphocytes) and subsets analyzed in patients and healthy controls using flow cytometry. The Mann-Whitney test was used to calculate the level of significance. Data are presented with medians and interquartile ranges. AAV: antineutrophil cytoplasmic autoantibody- (ANCA-) associated vasculitis; HBD: healthy blood donors; ns: not significant. ^a^*n* equals 101 for absolute values.

**Table 3:** Concentrations of IgA, IgG, and IgM in patients with AAV.

Concentration (g/L)	IgA	IgG	IgM
AAV patients (*n* = 92‐93)^a^	1.86 (1.32-2.75)	10.1 (8.46-12.9)	0.83 (0.60-1.33)
Diagnosis			
GPA (*n* = 56‐57)^a^	1.80 (1.40-2.75)	9.99 (8.60-12.9)	0.83 (0.61-1.42)
MPA (*n* = 33)	1.86 (1.31-2.67)	10.1 (7.66-12.6)	0.73 (0.58-1.23)
EGPA (*n* = 3)	3.74 (0.73-5.12)	11.7 (10.4-15.6)	1.51 (0.33-2.81)
Disease activity			
Active disease (*n* = 22‐23)^a^	1.99 (1.57-2.68)	9.79 (8.31-13.0)	0.72 (0.37-1.18)
Remission (*n* = 70)	1.86 (1.23-2.77)	10.4 (8.50-12.8)	0.83 (0.63-1.39)
Tendency to relapse			
Yes (*n* = 32‐33)^a^	1.80 (1.43-2.41)	9.88 (7.76-12.0)^∗^	0.98 (0.59-1.32)
No (*n* = 33)	2.02 (1.32-3.52)	11.4 (9.47-13.7)	0.75 (0.63-1.45)
Treatment			
AAV with ≥1 drugs (*n* = 68‐69)^a^	1.79 (1.38-2.44)	9.32 (7.75-12.4)^∗^	0.77 (0.54-1.30)
AAV no medication (*n* = 24)	2.82 (1.20-4.50)	12.0 (9.95-13.7)	0.96 (0.72-1.50)

Concentrations of IgA, IgG, and IgM in patients with AAV. Data are presented with medians and interquartile ranges. Ig: immunoglobulin; AAV: antineutrophil cytoplasmic autoantibody- (ANCA-) associated vasculitis; GPA: granulomatosis with polyangiitis; MPA: microscopic polyangiitis; EGPA: eosinophilic granulomatosis with polyangiitis. Reference range IgA 0.88-4.5 g/L, IgG 6.7-14.5 g/L, and IgM 0.27-2.1 g/L. ^a^For one patient, only IgG and IgM were measured. The Mann-Whitney test was used to calculate the level of significance. Subgroups of *n* < 5 were not included in the statistical analyses. ^∗^Denotes statistical difference as compared to comparator patient subgroup (listed below in the table).

**Table 4:** Frequencies of B cells and subsets in patients with AAV in active disease and remission.

Phenotype	Active disease (*n* = 24)	Remission (*n* = 82)	*p* value
BVAS3, median (range)	6 (2-26)	0 (0-1)	<0.0001
B cells (% of lymphocytes)	6.12 (2.97-9.70)	4.64 (3.15-8.98)	ns
B cells (10^6^/L)^a^	32.3 (17.6-84.5)	25.9 (15.0-66.4)	ns
B cell subsets (% of B cells)			
Naive	51.7 (35.6-58.3)	47.5 (30.5-63.6)	ns
Preswitch memory	4.93 (2.89-9.76)	6.71 (3.69-10.3)	ns
Switched memory	21.0 (14.2-29.5)	22.3 (13.6-33.1)	ns
Exhausted memory	19.8 (16.4-25.9)	21.5 (12.7-25.9)	ns
Transitional	1.35 (0.103-5.67)	0.675 (0.0600-2.81)	ns
Plasmablasts	0.755 (0.398-3.12)	0.905 (0.390-1.70)	ns
Activated B cells and subsets			
CD95^+^ B cells (% of B cells)	19.5 (9.50-33.8)	20.8 (12.3-32.8)	ns
CD95^+^ naive (% of naive)	2.79 (0.920-6.53)	3.47 (1.60-7.80)	ns
CD95^+^ preswitch memory (% of preswitch memory)	19.4 (11.3-39.5)	28.2 (16.8-38.7)	ns
CD95^+^ switched memory (% of switched memory)	59.1 (51.5-76.5)	61.0 (50.8-72.7)	ns
CD95^+^ exhausted memory (% of exhausted memory)	19.4 (11.2-33.0)	25.9 (16.3-35.6)	ns
Treatment, *n* (%)			
Prednisolone	17 (71)	39 (48)	
Azathioprine	6 (25)	27 (33)	
Methotrexate	1 (4)	8 (10)	
Mycophenolate mofetil	1 (4)	5 (6)	
Cyclophosphamide	5 (21)	6 (7)	
No medication	3 (13)	23 (28)	

Frequencies of B cells (CD19^+^ lymphocytes) and subsets analyzed in patients using flow cytometry. Active disease: BVAS3 ≥ 2; remission: BVAS3 ≤ 1. The Mann-Whitney test was used to calculate the level of significance. Data are presented with medians and interquartile ranges. AAV: antineutrophil cytoplasmic autoantibody- (ANCA-) associated vasculitis; BVAS3: Birmingham Vasculitis Activity Score version 3; ns: not significant. ^a^*n* equals 23 (active disease), 78 (remission) for absolute values.

**Table 5:** Frequencies of B cells and subsets in patients with AAV in remission with regard to tendency to relapse.

Phenotype	Tendency to relapse, remission (*n* = 29)	No tendency to relapse, remission (*n* = 36)	*p* value
B cells (% of lymphocytes)	3.51 (2.60-7.20)	6.53 (3.68-9.21)	0.0295
B cells (10^6^/L)^a^	18.6 (11.0-43.7)	45.4 (25.0-82.1)	0.0037
B cell subsets (% of B cells)			
Naive	47.3 (29.5-60.9)	53.7 (32.6-65.9)	ns
Preswitch memory	7.25 (4.40-10.6)	5.68 (3.51-11.2)	ns
Switched memory	23.9 (14.1-32.8)	18.7 (13.0-29.0)	ns
Exhausted memory	21.4 (11.3-25.2)	19.4 (12.8-26.0)	ns
Transitional	0.0900 (0.00-2.34)	1.10 (0.205-3.86)	0.0380
Plasmablasts	0.990 (0.265-2.61)	0.710 (0.383-1.48)	ns
Activated B cells and subsets			
CD95^+^ B cells (% of B cells)	25.3 (12.4-33.9)	16.7 (12.0-29.9)	ns
CD95^+^ naive (% of naive)	3.43 (1.75-9.46)	2.60 (1.24-5.36)	ns
CD95^+^ preswitch memory (% of preswitch memory)	29.9 (17.5-38.2)	21.4 (15.9-37.5)	ns
CD95^+^ switched memory (% of switched memory)	62.1 (52.5-71.8)	59.2 (50.2-72.5)	ns
CD95^+^ exhausted memory (% of exhausted memory)	30.8 (23.6-47.0)	22.3 (14.1-27.8)	0.0032
Treatment, *n* (%), dose			
Prednisolone, mg/day	19 (66) 7.50 (5.00-10.0)	13 (36) 5.00 (2.50-8.75)	ns
Azathioprine, mg/day	13 (45) 100 (75.0-100)	8 (22) 100 (62.5-150)	ns
Methotrexate, mg/week	5 (17) 25.0 (17.5-25.0)	2 (6) 18.8 (12.5-25.0)	
Mycophenolate mofetil, mg/day	2 (7) 2000 (2000-2000)	2 (6) 1125 (750-1500)	
Cyclophosphamide	2 (7)	2 (6)	
No medication	2 (7)	15 (42)	

Frequencies of B cells (CD19^+^ lymphocytes) and subsets analyzed in patients using flow cytometry. The Mann-Whitney test was used to calculate the level of significance. Data are presented with medians and interquartile ranges. AAV: antineutrophil cytoplasmic autoantibody- (ANCA-) associated vasculitis; ns: not significant. ^a^*n* equals 28 (tendency to relapse, remission), 33 (no tendency to relapse, remission) for absolute values.

## Data Availability

Raw data files from flow cytometry datasets used in the current study are available from the corresponding author on reasonable request.

## Supplementary Materials

Supplementary Materials Figure S1: gating strategy used to identify the B cell subpopulations. Table S1: frequencies of B cells and subsets in AAV patients with regard to medication.
