# Supplementary material for: Increased Frequencies of Switched Memory B Cells and Plasmablasts in Peripheral Blood from Patients with ANCA-Associated Vasculitis
Source: J Immunol Res. 2020 Nov 28;2020:8209737. doi: 10.1155/2020/8209737 (PMC7719539; doi:10.1155/2020/8209737)
Supplement: Supplementary Materials — Figure S1: gating strategy used to identify the B cell subpopulations. Table S1: frequencies of B cells and subsets in AAV patients with regard to medication. [file 8209737.f1.pdf]

Supplementary Materials

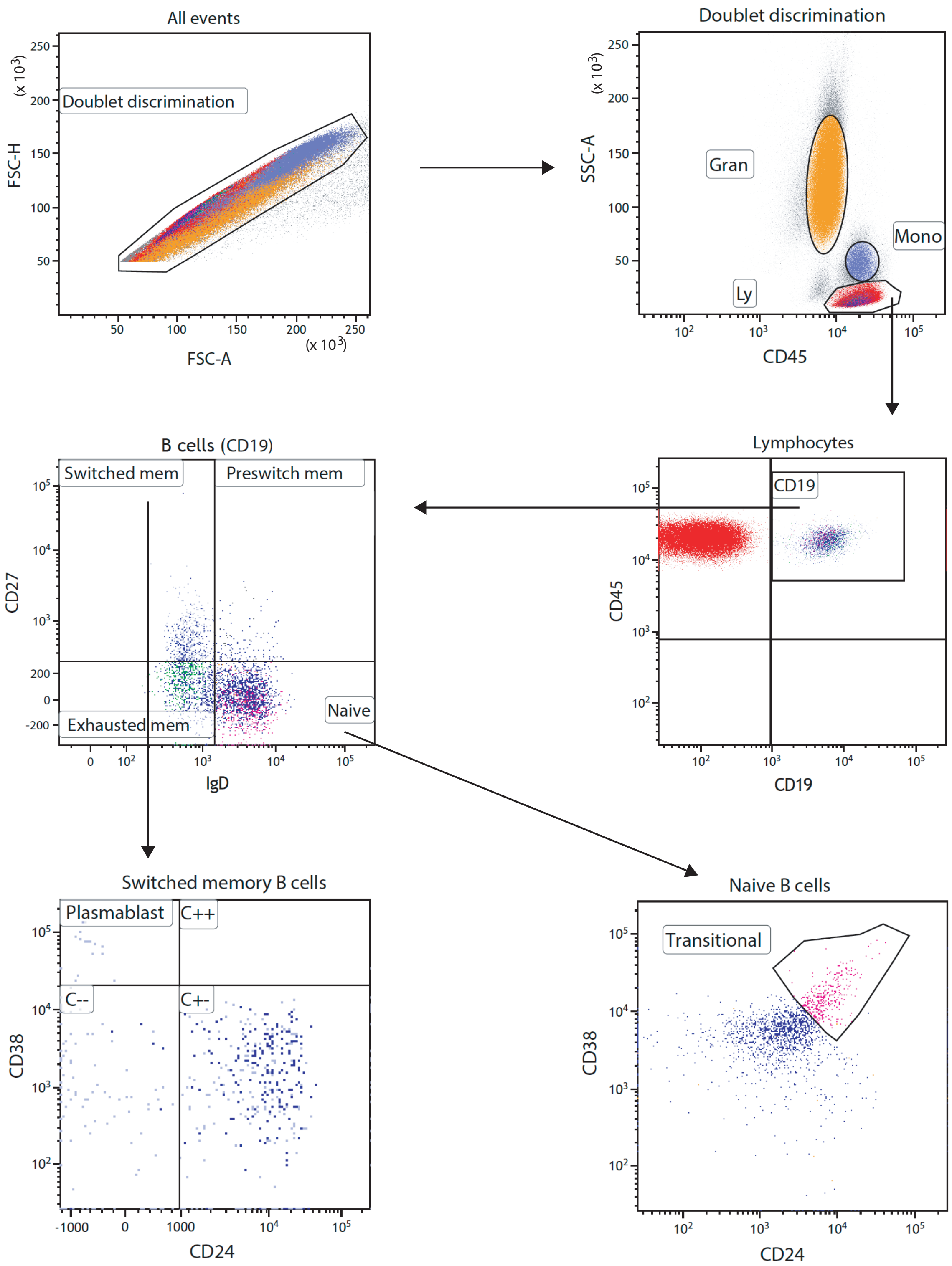

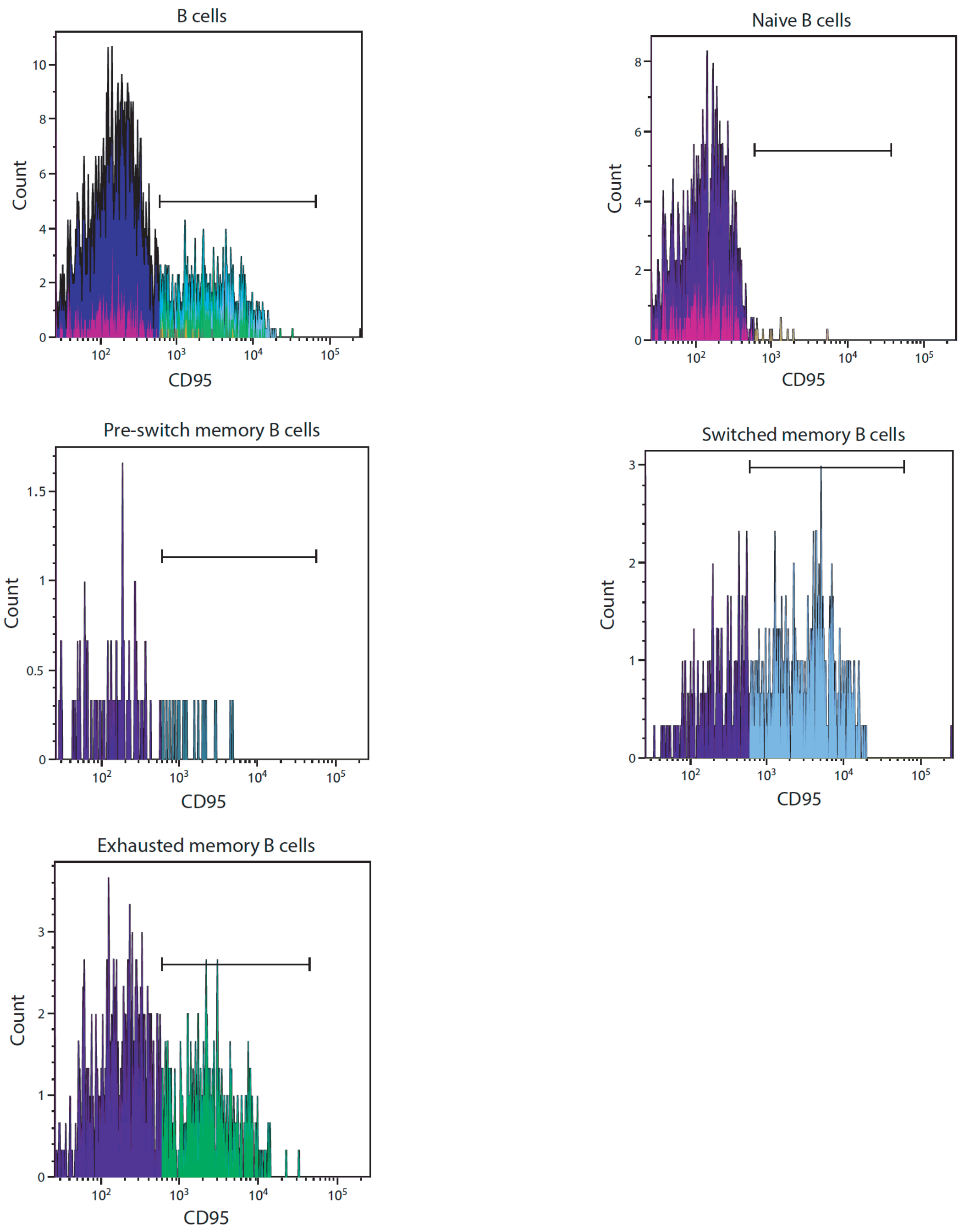

Supplementary Figure 1. Gating strategy for B cell subsets. A gate was first set on all events using the FSC-H/FSC-A plot for doublet discrimination. Then, a gate was set on lymphocytes using the SSC-A/CD45 plot. Within the lymphocytes CD19$^+$ B cells were gated using the CD45/CD19 plot. B cells were subsequently divided into naive B cells (CD19$^+$CD27$^-$IgD$^+$), pre-switch memory B cells (CD19$^+$CD27$^+$IgD$^+$), switched memory B cells (CD19$^+$CD27$^+$IgD$^-$) and exhausted memory B cells (CD19$^+$CD27$^-$IgD$^-$) using the CD27/IgD plot. Within the naive B cells, the transitional B cells (CD19$^+$CD27$^-$IgD$^+$CD24$^+$CD38^{++}) were gated using the CD38/CD24 plot. Within the switched memory B cells, the plasmablasts (CD19$^+$CD27$^+$IgD$^-$CD24$^-$CD38^{++}) were gated using the CD38/CD24 plot. Activated (CD95$^+$) B cells were gated within the respective population using the CD95 plot. The figure shows an example from a patient with anti-neutrophil cytoplasmic antibody (ANCA)-associated vasculitis (AAV).

Supplementary Table 1

Frequencies of B cells and subsets in patients with AAV with regard to medication

Phenotype	AAV with ≥1 drugs (n=80)	AAV no medication (n=26)	HBD (n=134)
**BVAS3, median (range)**	0 (0-26)	0 (0-21)	
**B cells (% of lymphocytes)**	4.29 (2.65-6.74)[a,b]	9.36 (7.39-13.7)	8.33 (5.98-11.6)
**B cells (10^6/L)***	21.5 (10.3-45.0)[a]	86.1 (40.3-153)	
**B cell subsets (% of B cells)**			
**Naive**	44.6 (27.9-56.4)[a,b]	65.3 (51.2-71.9)	56.7 (47.9-67.0)
**Pre-switch memory**	7.21 (4.40-10.7)[a]	4.29 (2.66-6.35)[c]	7.00 (4.65-10.2)
**Switched memory**	24.3 (17.3-34.4)[a,b]	9.94 (6.78-16.8)	16.5 (11.4-21.8)
**Exhausted memory**	21.1 (14.9-25.6)[b]	21.6 (11.4-26.2)	17.4 (12.0-21.6)
**Transitional**	0.235 (0.00500-1.74)[a,b]	3.65 (1.61-6.10)	4.44 (2.83-6.18)
**Plasmablasts**	1.00 (0.468-2.49)[b]	0.565 (0.348-1.13)[c]	0.320 (0.160-0.613)
**Activated B cells and subsets**			
**CD95⁺ B cells (% of B cells)**	25.6 (14.9-35.7)[a,b]	12.3 (6.88-17.1)	10.3 (7.24-15.5)
**CD95⁺ naive (% of naive)**	4.75 (1.98-9.38)[a,b]	1.16 (0.518-2.86)	1.02 (0.575-1.71)
**CD95⁺ pre-switch memory (% of pre-switch memory)**	31.1 (16.8-41.1)[a,b]	17.1 (9.93-28.9)	13.5 (9.07-19.8)
**CD95⁺ switched memory (% of switched memory)**	60.8 (52.2-72.9)[b]	56.4 (40.7-77.7)[c]	44.6 (36.0-51.9)
**CD95⁺ exhausted memory (% of exhausted memory)**	27.8 (18.0-38.6)[a,b]	16.1 (9.13-25.4)	14.0 (8.38-18.2)

Frequencies of B cells ($CD19^+$ lymphocytes) and subsets analysed in patients and healthy controls using flow cytometry. The patients were either medicated with ≥1 of the following medications; prednisolone, azathioprine, methotrexate, mycophenolate mofetil, cyclophosphamide, or none of them. Kruskal-Wallis with Dunn's multiple comparisons test was used to calculate level of significance. Mann-Whitney test was used to calculate level of significance for absolute values. Data are presented with medians and interquartile ranges. *AAV* anti-neutrophil cytoplasmic autoantibody (ANCA)-associated vasculitis, *HBD* healthy blood donors, *BVAS3* Birmingham Vasculitis Activity Score version 3. [a]Denotes significant difference between "AAV with ≥1" drugs and "AAV no medication". [b]Denotes significant difference between "AAV with ≥1" drugs and HBD. [c]Denotes significant difference between "AAV no medication" and HBD. [*]n equals 76 (AAV with ≥1 drugs), 25 (AAV no medication) for absolute values.
